# Parliament and the Professions

**Published:** 1920-03-06

**Authors:** 


### PARLIAMENT AND THE PROFESSIONS.

### Q.A.I.M.N.S.

Sir A. Williamson informed Mr. Nicholson that the rate of pay and pension for nurses of Queen Alexandra's Imperial Military Nursing Service is at present under consideration.

### Precautionary Measures Against Influenza.

Dr. Addison stated that there had been a slight increase in the number of deaths from influenza during recent weeks, and a few significant outbreaks in schools and other institutions, but otherwise there is no evidence at present of unusual prevalence of the disease in this country comparable to the definite new waves of influenza which are occurring in American cities and on the Continent. The Ministry had placed itself in a position closely to watch the occurrence of the disease at home and abroad and to observe the progress of an epidemic. A vaccine is issued on demand to Medical Officers of Health for distribution free of charge among medical practitioners within their districts, and other preventive measures had been taken.

### Taxation of Motor Invalid Chairs.

The Chancellor of the Exchequer stated that under the existing law mechanically-propelled invalid chairs, if used upon the public highway, are liable to motor-car licence duty. The whole question of the taxation of mechanically propelled vehicles is at present being considered by a Departmental Committee appointed by the Minister of Transport, and the question of the exemption of this particular class of vehicle must await the Report.

